# Advancing Normal Tissue Complication Probability Modeling with Supervised Contrastive Learning for Predicting Osteoradionecrosis

**DOI:** 10.1145/3748522.3779983

**Published:** 2026-06-09

**Authors:** Eric Ababio Anyimadu, Xinhua Zhang, Clifton David Fuller, G. Elisabeta Marai, Guadalupe Canahuate

**Affiliations:** Electrical and Computer Engineering, University of Iowa, Iowa City, Iowa, USA; Computer Science, University of Illinois Chicago, Chicago, Illinois, USA; Radiation Oncology, University of Texas MD Anderson Cancer Center, Houston, Texas, USA; Computer Science, University of Illinois Chicago, Chicago, Illinois, USA; Electrical and Computer Engineering, University of Iowa, Iowa City, Iowa, USA

**Keywords:** Contrastive Learning, Normal Tissue Complication, Osteoradionecrosis, Dose-Volume Histogram

## Abstract

Normal tissue complication probability (NTCP) modeling using dose–volume histograms (DVHs) is fundamentally challenged by high dimensionality, severe multicollinearity, and substantial overlap between DVH profiles of patients with differing toxicity outcomes, limiting the effectiveness of classical classification approaches. We introduce SC–NTCP, a supervised contrastive learning framework that transforms DVH data into a compact, separable latent representation optimized for predicting osteoradionecrosis (ORN) in head and neck cancer patients. Rather than relying on raw, high-dimensional DVH features, SC–NTCP explicitly maximizes intra-class similarity and inter-class separability within the embedding space, enabling more accurate downstream classification. Using a cohort of head and neck cancer patients, we benchmarked SC–NTCP against logistic regression, support vector machines, multilayer perceptrons, and convolutional neural networks. SC–NTCP demonstrated superior discrimination (AUC = 0.77), improved calibration, and enhanced interpretability via gradient-based feature attribution, while the integration of clinical covariates further augmented predictive performance. By addressing the inherent limitations of DVH data, SC–NTCP offers a principled and interpretable approach for robust radiation toxicity prediction, with the potential to inform personalized treatment planning and improve clinical outcomes.

## 1 Introduction

Osteoradionecrosis (ORN) of the mandible is a severe and debilitating late complication of radiotherapy in patients with head and neck cancer (HNC). It is characterized by non-healing bone or persistent mucosal breakdown lasting for several months [9, 22]. The onset of ORN arises from a multifactorial interplay among radiation dose, clinical conditions, and patient-specific characteristics [14, 24]. Although its reported incidence varies between 1–16% [11, 24], the condition can lead to severe pain, dysesthesia, dysgeusia, and impaired mastication, which profoundly diminishes patients’ quality of life [24].

The often prolonged treatment course of ORN, which ranges from conservative management to extensive surgical resection with sometimes unsuccessful outcomes, underscores that prevention through meticulous radiation therapy planning remains the most effective clinical strategy for managing this complication [14, 22, 24]. Therefore, reliable risk assessment prior to treatment delivery is essential for personalizing dose constraints and optimizing therapeutic decisions [1, 23].

Normal tissue complication probability (NTCP) models provide a quantitative framework for balancing tumor control with the protection of normal tissues or organs at risk (OARs) by estimating the likelihood of radiation-induced toxicities such as ORN [17]. These models often rely on Dose-Volume Histograms (DVHs), which condense the three-dimensional dose distribution within an OAR into a two-dimensional curve representing the fraction of normal tissue volume receiving a given radiation dose level [26, 28]. A robust NTCP model can guide the establishment of dose constraints, support modality selection, and identify high-risk patients who may benefit from closer clinical surveillance [12, 24].

However, developing effective NTCP models for ORN from DVH data faces considerable challenges. The dose-volume features are inherently high-dimensional and exhibit severe multicollinearity, undermining the stability of traditional machine learning models [13, 24]. Furthermore, DVH curves from patients who develop ORN show extensive overlap with those who do not, indicating poor inherent separability in the original feature space [13]. This is consistent with the vascular pathogenesis of ORN and the inability of DVHs to capture fine-scale spatial dose heterogeneity [13].

To address these limitations, many studies have simplified DVHs into univariate summary metrics such as the mean dose or D50% (the dose delivered to 50% of the OAR volume) [24]. Although convenient, this reduction discards critical structural information, and clinically distinct DVHs associated with different outcomes can collapse to identical summary values. This shortcoming, initially observed in classical single-value models such as the Lyman–Kutcher-Burman (LKB) formulation [17], continues to constrain modern data-driven approaches.

For instance, Van Dijk et al. (2021) [24] employed univariable logistic regression across individual dose parameters, identifying D30% as the strongest predictor of ORN. While informative, such methods fail to exploit the full multivariate structure of the DVH or learn representations explicitly optimized for class separation. Moreover, these reductions and simplifications may not fully reflect clinical decision-making, as radiotherapy planning typically considers the entire DVH rather than a single dose parameter when balancing competing constraints [17, 24]. This highlights both the inadequacy of single-value summaries and the need for more expressive modeling paradigms.

More recently, Hosseinian et al. (2024) [13] sought to utilize the complete DVH information by applying K-means clustering to capture underlying structural patterns, followed by a soft-margin SVM for ORN risk stratification. Although more comprehensive, their findings reinforced the persistent challenge that the DVH of affected and unaffected patients exhibit substantial overlap, leading to less distinct cluster boundaries and reduced model robustness.

Contrastive learning, on the other hand, offers a powerful alternative for modeling data characterized by poor separability, multicollinearity, and high dimensionality. Originally developed for self-supervised learning, this paradigm learns latent embeddings by pulling similar samples closer together and pushing dissimilar ones apart within a latent space [16]. The approach has since been extended to supervised settings, where class labels are explicitly leveraged to refine the contrastive process through similarity measures [15]. This makes supervised contrastive learning particularly well-suited for optimizing data separability in domains where intrinsic class overlap poses a major challenge.

To address the existing gap in NTCP modeling using DVH data, we propose a novel framework based on supervised contrastive learning, termed *SC–NTCP*, to derive discriminative latent representations of DVH features for ORN risk prediction.

We hypothesize that this approach will mitigate the limitations of traditional NTCP models by transforming the inherently non-separable DVH data into an embedding space where a downstream classifier can achieve improved discrimination between ORN and non-ORN cases.

Experiments were performed on a large, publicly available DVH dataset of HNC patients collected at M.D. Anderson Cancer Center. Our analysis demonstrates that the proposed contrastive learning approach not only enhances predictive accuracy but also provides meaningful interpretability by identifying the key dose levels associated with ORN development.

In summary, the contributions of this paper are as follows:

- We propose, to the best of our knowledge, the first application of supervised contrastive learning to develop an interpretable NTCP model for predicting ORN from DVH data.
- Through t-SNE plots, we illustrate how our approach can enhance the separability of the originally non-separable DVH data, producing a latent space with more distinct class clusters.
- We demonstrate that a simple logistic regression classifier trained on the learned embeddings significantly outperforms conventional models applied directly to raw DVH features, as measured by the Area Under the ROC Curve (AUC).

## 2 Related Works

The success of contrastive learning in computer vision has inspired growing interest in its application to medical imaging and healthcare analytics, domains often characterized by high-dimensional data, complex feature relationships, and limited labeled samples [8]. In medical imaging, supervised contrastive learning has demonstrated substantial improvements in classification and segmentation tasks, including chest X-ray interpretation [7] and histopathology image analysis in semi-supervised settings [20]. These approaches outperform conventional deep learning methods by effectively leveraging the intrinsic structure and similarities inherent in medical data.

Beyond imaging, contrastive learning frameworks have been extended to structured clinical and temporal data. Supervised contrastive methods have been applied to electronic health records for patient risk prediction [29], to survival analysis [19], and to genomics for learning low-dimensional embeddings of biological markers [21]. A key strength of these approaches lies in their ability to learn latent representations where clinically similar patients are embedded closer together, capturing nuanced relationships that go beyond simple feature-level similarity.

Despite this growing body of work, contrastive learning has not yet been explored in the context of NTCP modeling, particularly for radiation-induced toxicities such as ORN using DVH data.

In this study, we present the application of supervised contrastive learning to NTCP modeling for ORN prediction. Our framework learns clinically meaningful representations directly from full, high-dimensional DVH curves and is explicitly designed to address the limitations of traditional NTCP approaches by optimizing the embedding space for maximal inter-class separability and intra-class cohesion.

### 3 Problem Formulation

Consider a cohort of N patients, each with a cumulative dose discretized at k volume percentages, forming the design matrix:

(1) $$X={\left[x_{1}\;\ldots \;x_{N}\right]}^{⊤}\in R^{N\times k},$$

where each row $x_{i}=\left[D_{i}\left(v_{1}\right),\ldots ,D_{i}\left(v_{k}\right)\right]\in R^{k}$ represents the DVH of the i-th patient, with $D_{i}\left(v_{k}\right)$ denoting the dose D(Gy) delivered to at least $v_{k}\%$ of the mandible.

Each patient is further associated with a binary clinical outcome:

(2) $$y_{i}\in \{0,1\},\;y_{i}=\left\{\begin{array}{ll} 1 & \text{if ORN is present},\\ 0 & \text{otherwise}. \end{array}\right.$$

The objective is to learn a mapping:

(3) $$f.:R^{k}\to R^{d},\;d\neq k,$$

that embeds each DVH $x_{i}$ into a d-dimensional latent space optimized to enforce intra-class compactness, where patients with the same outcome are mapped close together, and inter-class separation, where patients with different outcomes are distinctly separated. This learned representation is used for improved performance in downstream classification tasks.

## 4 Proposed Method

The proposed *SC–NTCP* framework builds upon the supervised contrastive learning paradigm implemented by Khosla et al. [15], which extends self-supervised contrastive learning principles to fully supervised settings.

**Figure 1:**
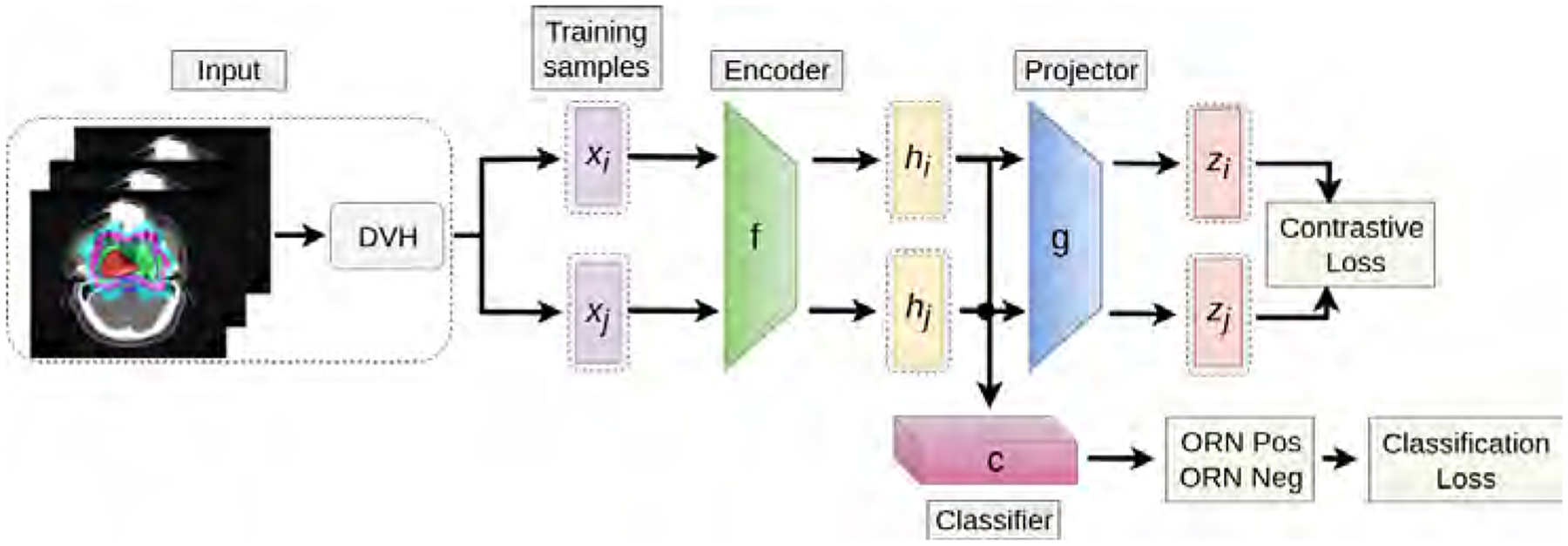
Overview of the proposed SC–NTCP framework.

As illustrated in Figure 1, the proposed framework is composed of three main modules: an encoder, a projector, and a classifier. We denote these components as $f_{\theta },g_{\phi }$, and $c_{\psi }$, representing the encoder, projector, and classifier networks, parameterized by learnable weights θ,ϕ, and ψ, respectively.

During training, these modules are jointly optimized using a combination of supervised contrastive and classification losses. This joint optimization guides the encoder to learn representations that are simultaneously discriminative for contrastive learning and informative for the downstream ORN classification task [6].

Once training is complete, the encoder is frozen, and the resulting embeddings are used to train a downstream classification model supervised with the corresponding ORN labels.

For a new patient, the DVH is first encoded using the frozen encoder, and the resulting embedding is passed to the pre-trained downstream classification model to generate predictions.

A detailed description of the proposed framework is provided below.

### 4.1 Encoder

The encoder $f_{\theta }$ maps each DVH $x_{i}\in R^{k}$ to a latent representation:

(4) $$h_{i}=f_{\theta }\left(x_{i}\right),\;h_{i}\in R^{d},$$

where d represents the dimensionality of the learned latent feature space.

We implement $f_{\theta }$ as a convolutional neural network (CNN) that extracts compact and discriminative latent representations from the DVH inputs.

### 4.2 Projector

The projector $g_{\phi }$ transforms embeddings $h_{i}$ into a space optimized for contrastive learning:

(5) $$z_{i}=g_{\phi }\left(h_{i}\right),\;z_{i}\in R^{d^{\prime }},$$

where $d^{\prime }$ denotes the dimensionality of the projection space.

The projector $g_{\phi }$ is implemented as a multilayer perceptron (MLP) that maps the latent embeddings to a lower-dimensional space optimized for contrastive learning.

### 4.3 Classifier

The classifier $c_{\psi }$ produces predicted probabilities for ORN occurrence using the encoder output $h_{i}$:

(6) $${\overset{ˆ}{y}}_{i}=c_{\psi }\left(h_{i}\right),\;{\overset{ˆ}{y}}_{i}\in \left[0,1\right],$$

The classifier $c_{\psi }$ is implemented as an MLP that maps the latent embeddings to a scalar output through successive transformations for binary prediction.

### 4.4 Loss Functions

#### Contrastive Loss.

The supervised contrastive loss is computed using cosine similarity between normalized projector outputs [8]:

(7) $${ℒ}_{\text{contrast}}=\frac{1}{B}\sum_{i=1}^{B}\frac{-1}{\left|P(i)\right|}\sum_{p\in P(i)}\text{log}\frac{\text{exp}\left(z_{i}\cdot z_{p}/\tau \right)}{\sum_{a\in A\left(i\right)}\text{exp}\left(z_{i}\cdot z_{a}/\tau \right)},$$

where $z_{i}$ is the anchor embedding, $z_{p}$ is a positive sample embedding sharing the same label as the anchor, and $z_{a}$ are the embeddings of all other samples in the batch of size B,τ is a temperature hyperparameter, A(i)={1,2,…,B}∖{i} represents all batch indices except i, and $P(i)=\left\{p\in A(i)|y_{p}=y_{i}\right\}$ denotes positive samples sharing the same label as sample i.

#### Classification Loss.

The classifier is trained with binary cross-entropy loss:

(8) $${ℒ}_{\text{class}}=-\frac{1}{B}\sum_{i=1}^{B}\left[y_{i}\;\text{log}\;{\overset{ˆ}{y}}_{i}+\left(1-y_{i}\right)\;\text{log}\left(1-{\overset{ˆ}{y}}_{i}\right)\right],$$

where $y_{i}$ and ${\overset{ˆ}{y}}_{i}$ are the true label and predicted probability for sample i, respectively.

### 4.5 Training Objective

The total training loss combines the classification and contrastive losses:

(9) $${ℒ}_{\text{total}}=\left(1-\gamma \right)\cdot {ℒ}_{\text{class}}+\gamma \cdot {ℒ}_{\text{contrast}},$$

where γ∈[0,1] is a hyperparameter that balances the relative contribution of the contrastive term. The objective during the training phase is to minimize the total loss ${ℒ}_{\text{total}}$.

### 4.6 Inference

For an unseen patient DVH data $x^{*}$, inference is performed in two stages. First, the frozen encoder is used to extract the embedding:

(10) $$h^{*}=f_{\theta }\left(x^{*}\right),$$

Then, a logistic regression classifier trained on the learned embeddings from the training set produces the predicted probability of ORN:

(11) $${\overset{ˆ}{y}}^{*}=\sigma \left(w^{⊤}h^{*}+b\right),$$

where σ is the sigmoid function, and w and b are the learned weights and bias of the logistic regression model. A binary prediction is then obtained using a threshold of 0.5:

(12) $${\overset{ˆ}{y}}_{\text{binary}}^{*}=\left\{\begin{array}{ll} 1, & \text{if}\;{\overset{ˆ}{y}}^{*}>0.5,\\ 0, & \text{otherwise.} \end{array}\right.$$

The use of a linear classifier for inference highlights the quality and separability of the learned representations, demonstrating that the encoder produces embeddings that are linearly separable with respect to the ORN outcome.

### 4.7 Implementation Details

The encoder is a 1D CNN with five convolutional blocks, chosen via ablation and grid search to balance capacity and generalization (deeper models overfit; shallower models underfit). Each block contains Conv1D layers with increasing filters (8, 16, 32, 64, 128), batch normalization, ReLU activation, and dropout (0.2). Progressive filter expansion enables hierarchical feature learning, capturing local dose variations in early layers and global DVH patterns in deeper layers. Global average pooling provides translation invariance and reduces overfitting, followed by a fully connected layer producing a d-dimensional embedding (d=16), selected to balance expressiveness, efficiency, and overfitting risk.

A three-layer MLP projection head maps encoder embeddings to a contrastive space, applying fully connected layers with ReLU and dropout (0.2) to produce a $d^{\prime }$-dimensional output $\left(d^{\prime }=8\right)$. This depth balances expressiveness and training stability. The projector output is L1-normalized before the contrastive loss computation to enforce uniform distribution on the unit hypersphere and prevent magnitude-driven clustering.

The training classifier is a three-layer MLP (256 and 128 hidden units, ReLU, dropout 0.2) with a sigmoid output for binary ORN prediction, implemented as a separate head to decouple representation learning from task-specific prediction. The contrastive–classification loss weight was set to γ=0.8 via grid search over [0.1, 0.9] in 0.1 increments, optimizing validation AUC and embedding quality; higher values yielded improved latent space separation, particularly under class imbalance.

At inference, a L1-regularized logistic regression classifier (C=1, selected via nested cross-validation, liblinear solver, class-balanced) is trained on frozen embeddings to assess linear separability and embedding quality.

Class imbalance (~14% ORN positive) is addressed using weighted random sampling (inverse-frequency class weights) to construct balanced mini-batches, improving positive-pair availability and hard negative diversity for contrastive learning. DVH features are z-score normalized using training set statistics.

Models are trained with Adam ($\eta =10^{-3}$, weight decay 10^−3^) and cosine annealing, using batch size B=64 for up to 100 epochs, with early stopping based on validation AUC (patience = 10). Also, 10% of the training set is reserved for stratified internal validation. The checkpoint with the highest validation AUC is used to generate embeddings for the final classifier. Model selection prioritized cross-fold stability over single-split performance to enhance robustness and clinical generalizability.

### 4.8 Interpretability Analysis

#### 4.8.1 DVH and Embedding Representations.

To examine the class separability of the embeddings relative to the original DVH features, we use t-SNE to project both into two-dimensional representations for training and testing data, enabling a visual assessment of how well the contrastive framework organizes the latent space.

#### 4.8.2 Feature Importance Analysis via Gradient Attribution.

To quantify the importance of each dose-volume feature in the learned embedding space, we compute the *average gradient magnitude* (AGM) [2]. For a trained encoder $f_{\theta }:R^{k}\to R^{d}$ and a set of M test samples, the AGM for feature j is:

(13) $${\text{AGM}}_{j}=\frac{1}{M}\sum_{i=1}^{M}\left|\frac{\partial {\left\|h_{i}\right\|}_{2}}{\partial x_{i,j}}\right|,$$

where $h_{i}=f_{\theta }\left(x_{i}\right)$ is the embedding for sample $i,{\left\|h_{i}\right\|}_{2}=\sqrt{\sum_{j=1}^{d}h_{i,j}^{2}}$ is its L2 norm, and $x_{i,j}$ is the j-th input feature. This metric quantifies the sensitivity of the embedding magnitude to perturbations in each input feature, identifying which dose levels most strongly influence the learned representation. Gradients are computed via automatic differentiation, and we analyze M=100 randomly selected test samples for computational efficiency.

## 5 Experimental Setup

To assess the performance of the proposed *SC–NTCP* framework, we systematically evaluate two of its variants derived based on the data used for the classification task and compare their performance against standard baseline classification models.

### 5.1 *SC–NTCP* Model Variants

The proposed *SC–NTCP* framework derives dose embeddings from the DVH representation, which are then used for classification in unseen patients. Certain clinical factors, specifically pre-radiation dental extraction or edentulous status and smoking history (current or former) have been consistently identified as significant predictors or risk factors for ORN in prior studies [14, 18, 24]. Given the established importance of these variables in patient management, it is natural to incorporate them alongside the learned DVH embeddings. In the integrated model, *SC–NTCP*, the clinical features are concatenated with the contrastively derived embeddings prior to training the final logistic regression classifier, allowing the model to leverage both dosimetric and patient-specific risk information. We also compare performance to the same model excluding clinical features, *SC–NTCP* (*No clinical*), to demonstrate the added value of including essential patient-specific risk factors. Both variations use the same contrastively derived DVH embeddings.

### 5.2 Baseline Models for Comparison

To assess the added value of the contrastive embeddings in the *SC–NTCP* model, we further compare it against several baseline approaches that are trained on the DVH features and clinical variables and do not use the learned embeddings. These baseline models are selected to span a range of commonly used machine learning and neural network methods, providing a context to evaluate the benefit of the embedding-based framework. All models use the same DVH features and clinical variables as in *SC–NTCP*. For neural network baselines, balanced mini-batch sampling is employed to mitigate class imbalance.

#### Logistic Regression (LR).

A linear baseline using the same configuration as the logistic regression classifier in *SC–NTCP*.

#### Multi-Layer Perceptron (MLP).

A feedforward neural network with an architecture that mirrors the MLP used within *SC–NTCP*.

#### Convolutional Neural Network (CNN).

A 1D CNN that shares the same design principles as the encoder–classifier pathway in *SC–NTCP*.

#### Support Vector Machine (SVM).

An SVM with an RBF kernel, a regularization parameter C=1.0, class balancing, and probability estimates enabled for AUC evaluation.

These baseline models provide a spectrum of linear and nonlinear methods trained directly on DVH and clinical features. Comparing their performance to *SC–NTCP* highlights the benefits of incorporating contrastive embeddings to capture more informative representations of the dose distribution.

### 5.3 Evaluation

Statistical validation is performed using five-fold stratified cross-validation to ensure the robustness of the analysis. The performance of all classification models is evaluated using three metrics: Area Under the Receiver Operating Characteristic Curve (AUC-ROC), Accuracy (ACC), and F1 Score. For each metric, we report the mean value and 95% confidence interval across the five test folds.

## 6 Results

### 6.1 Data

We used the dataset compiled by van Dijk et al. [24], which includes anonymized clinical records and mandibular DVHs from 1,259 HNC patients treated with radiotherapy, with or without surgery and/or chemotherapy, at The University of Texas MD Anderson Cancer Center between 2005 and 2015 under IRB approval (RCR030800). The dataset contains detailed clinical variables such as age, sex, cancer subsite, T-stage, N-stage, preradiation dental extraction status (within six weeks before treatment), smoking status and pack-years, treatment intent (definitive or postoperative), and mandible bone volume. DVH data include D2%, D5%–D95% in 5% increments, D97%, D98%, and D99%.

Most of the patients, 1,086 (86.3%), remained ORN-free during at least 12 months after therapy, while 173 (13.7%) developed ORN (Grades I–IV). The median follow-up duration was 57 months (range: 12–174 months). Most patients were male (83%), and the most common diagnosis was oropharyngeal cancer (66%), followed by oral cavity (15%) and laryngeal cancer (13%). Additional details are presented in Table 1, and a comprehensive description of the cohort can be found in van Dijk et al. [24]. The dataset is also publicly available at https://doi.org/10.6084/m9.figshare.13568207.

**Figure 2:**
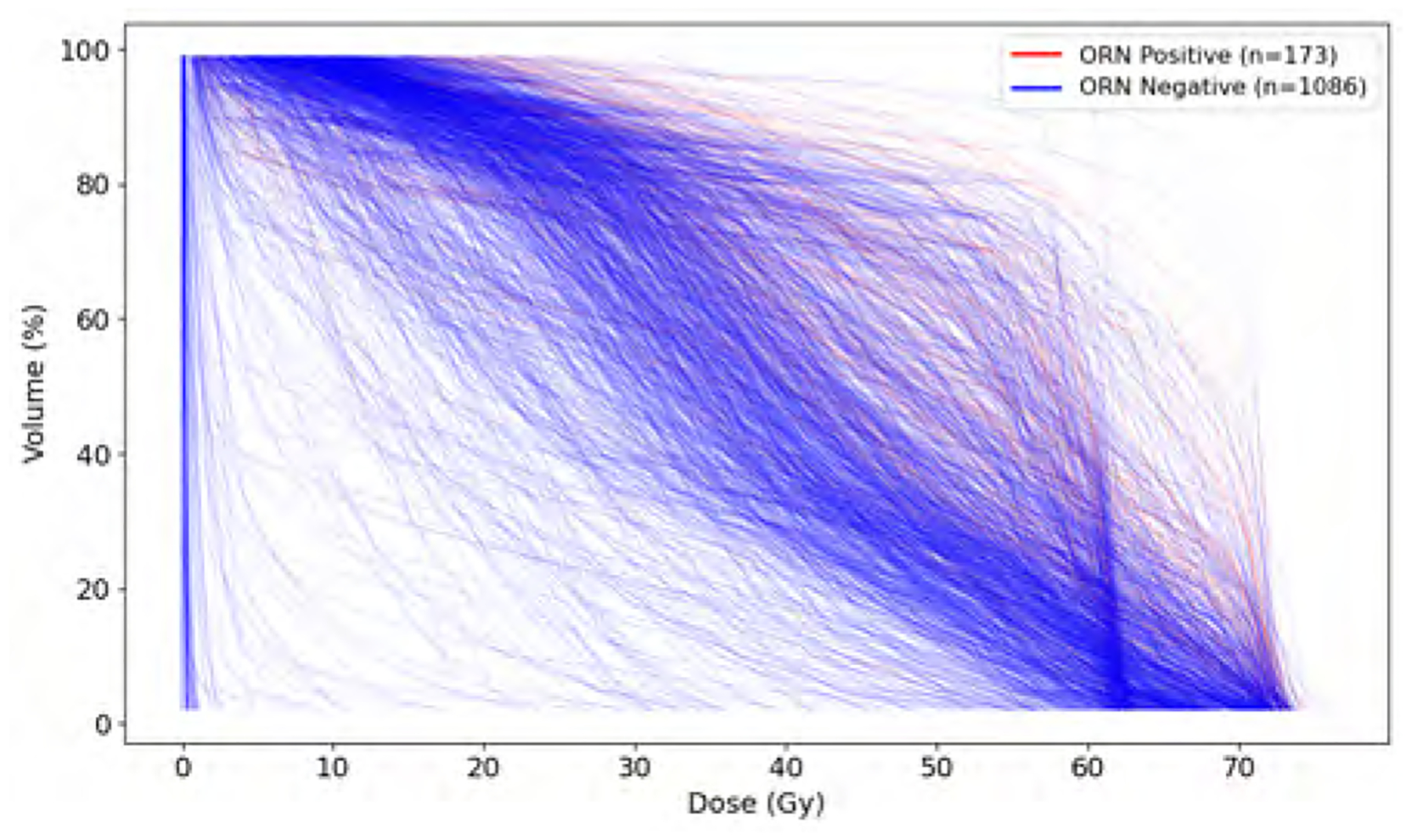
Dose–volume curves for individual patients, showing mandible dose (Gy) on the x-axis versus relative volume (%) on the y-axis. Curves are colored by ORN outcomes (Negative and Positive)

Figure 2 presents the DVH curves for all patients in the cohort, stratified by ORN status, to highlight the degree of separability between the classes.

### 6.2 Performance Comparison

**Figure 3:**
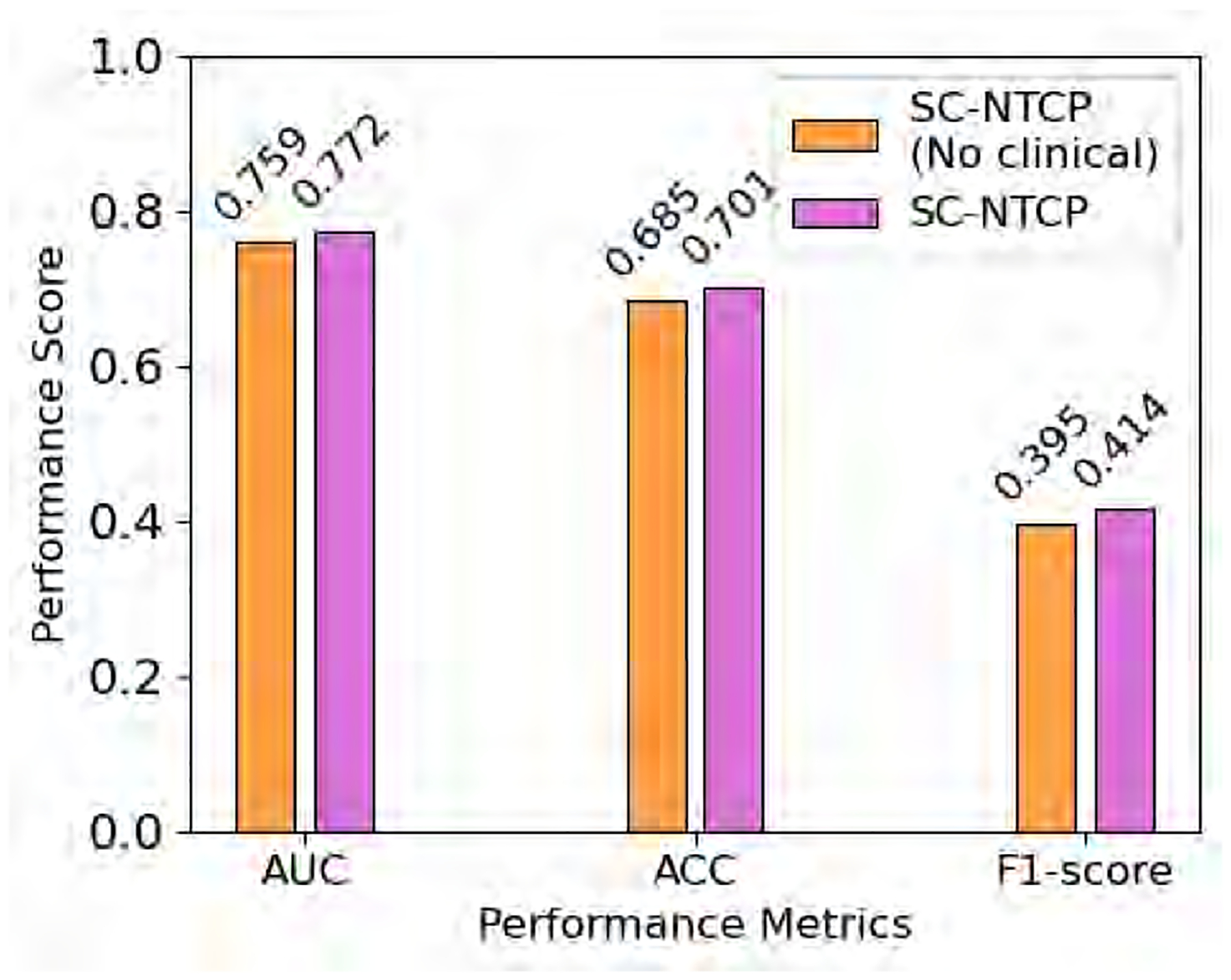
Performance Comparison of the Proposed *SC–NTCP* Model Variants Using DVH and Clinical Variables and No Clinical Input for the Classification Task

Figure 3 presents a performance comparison between the *SC–NTCP* variant that predicts ORN using contrastively derived DVH embeddings combined with clinical variables and the variant that excludes clinical inputs. Across all evaluation metrics (AUC, ACC, and F1-score), the integrated model that incorporates both DVH and clinical features consistently outperforms the model relying solely on DVH information.

**Figure 4:**
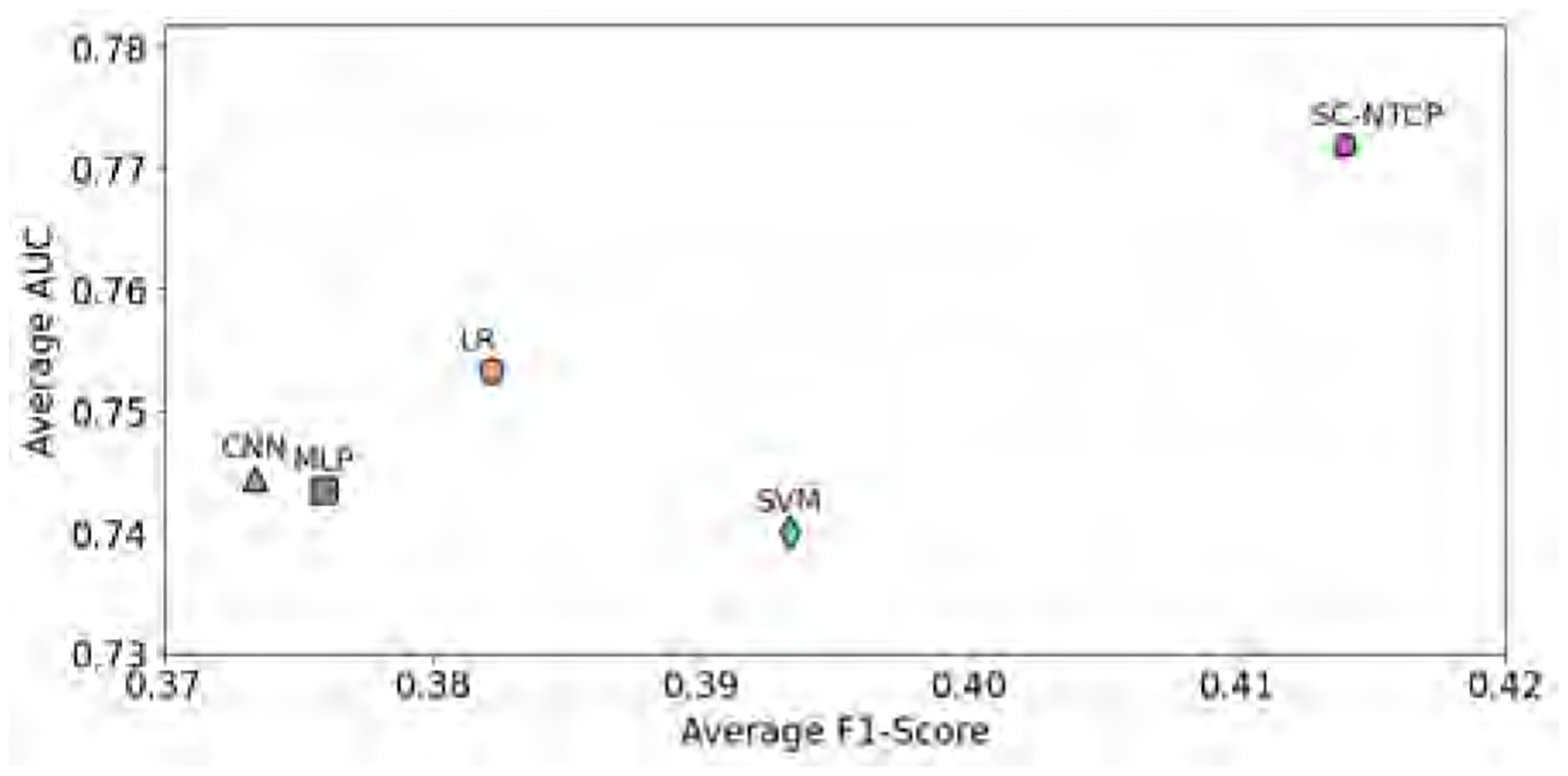
Performance Comparison of the Proposed *SC–NTCP* Variants and Baseline Models in terms of Average AUC and F1-Score

Figure 4 presents the performance comparison between the proposed *SC–NTCP* model and the LR, MLP, CNN, and SVM baselines. The plot illustrates the relationship between the average AUC and average F1-score, emphasizing the relative performance of the models. As shown, the *SC–NTCP* model demonstrates superior performance across both metrics compared to all baseline approaches. Table 2 further details the comparative performance, reporting the average AUC, ACC, and F1-score values along with their corresponding 95% confidence intervals. The *SC–NTCP* model achieved the best overall results, attaining the highest average AUC (0.7718), F1-score (0.4140), and accuracy (0.7010).

### 6.3 Interpretability Analysis

#### 6.3.1 DVH and Embedding Representations.

**Figure 5:**
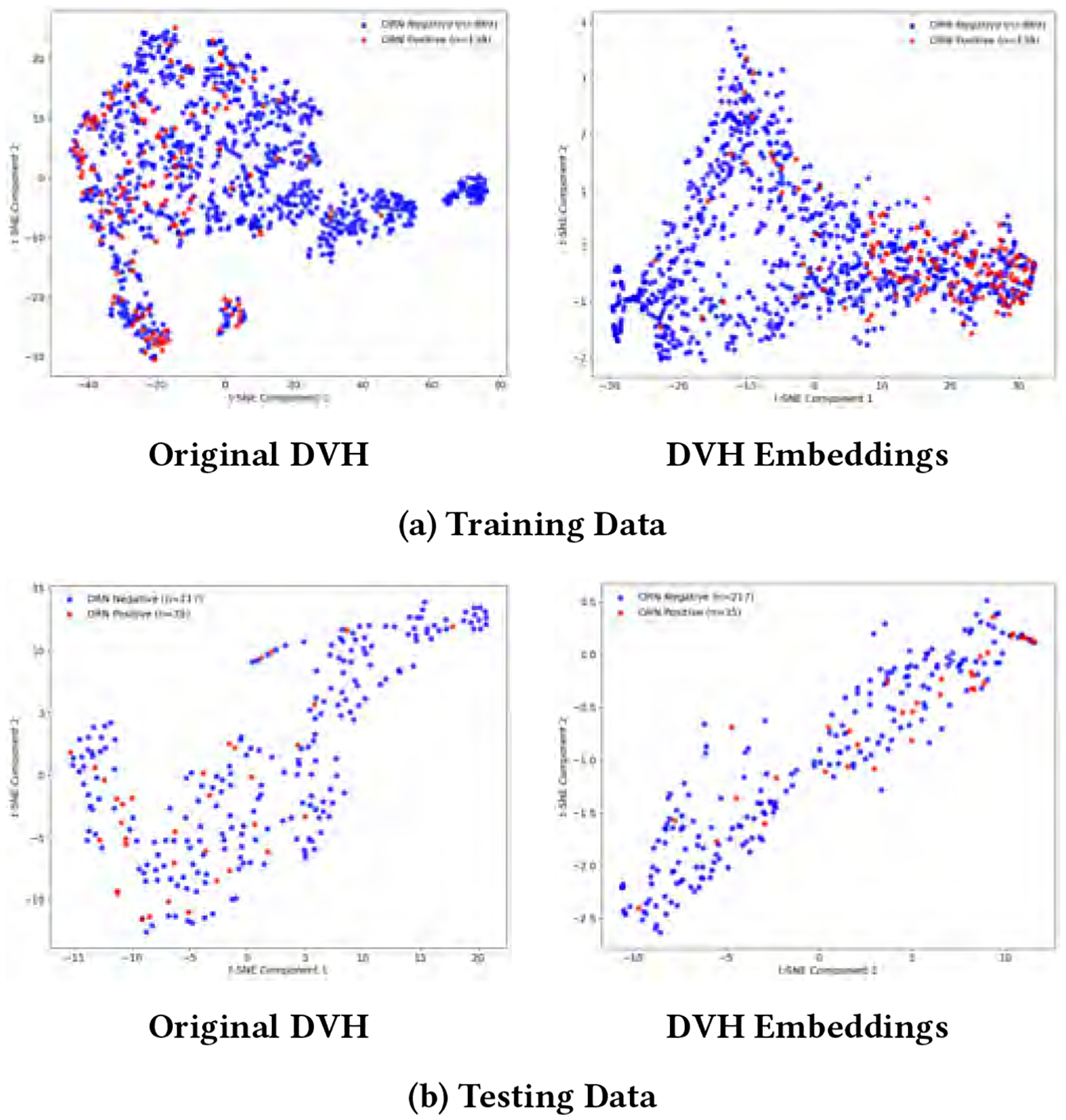
t-SNE plots of the Original DVH (left) and the *SC–NTCP* Derived Embeddings (right) Across the Training Data (a) and the Testing Data (b) Stratified by ORN Status.

Figures 5a and 5b present t-SNE projections of the original DVH features and the embeddings derived from the *SC–NTCP* model for the training and testing datasets, respectively. In each figure, the original DVH is shown on the left, and the corresponding embeddings are shown on the right, with points colored by ORN status. The embeddings exhibit improved separation between ORN-positive and ORN-negative cases compared to the original DVH features, reflecting the ability of the contrastive framework to organize the latent space more meaningfully. This improvement is particularly evident in the testing dataset, which contains fewer samples and also suggests that the learned embeddings generalize well to unseen data.

#### 6.3.2 Feature Importance Analysis.

**Figure 6:**
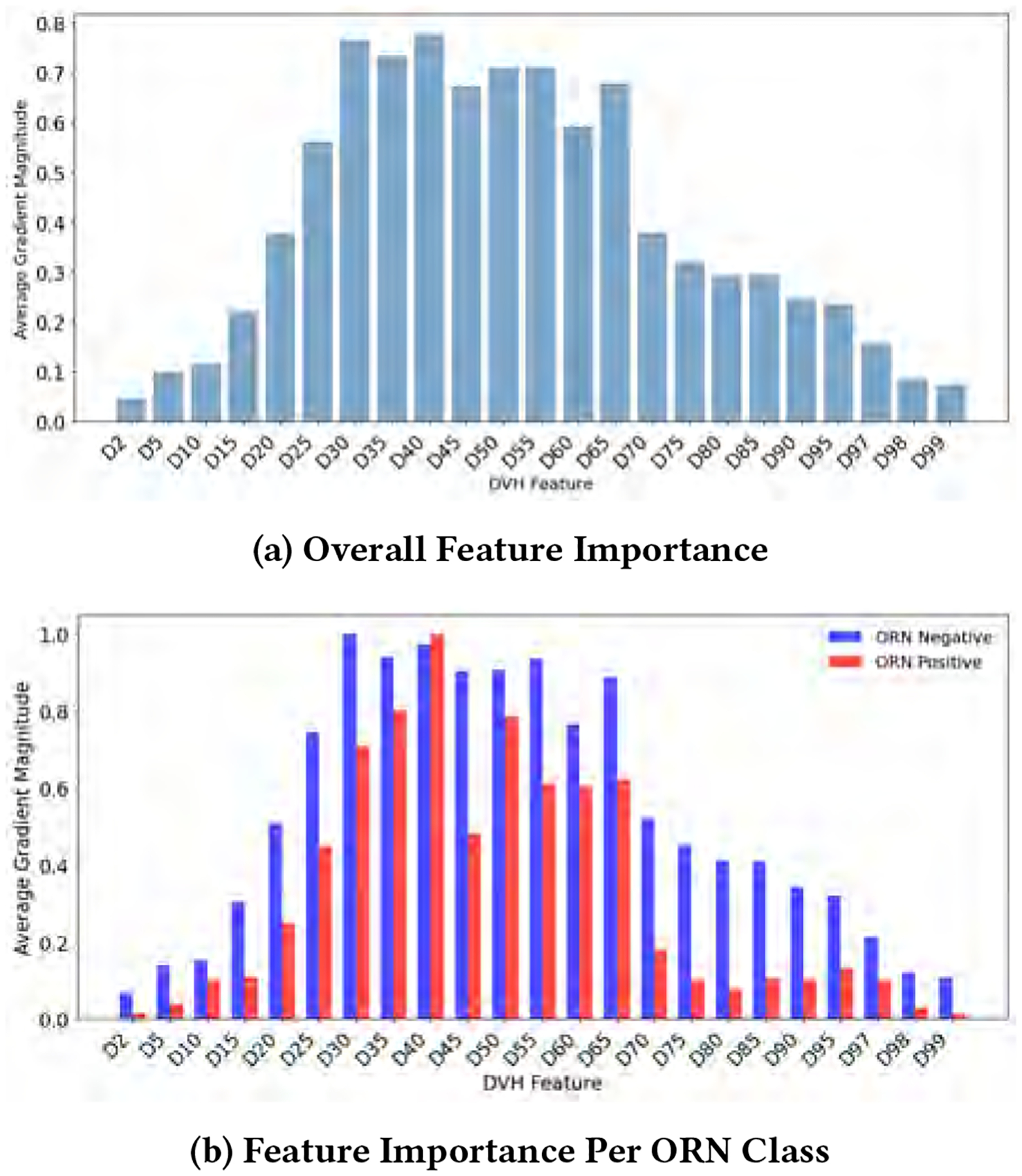
Feature Importance of the Dose-volume features in the Learned Embedding Space Determined Using Average Gradient Magnitude (AGM)

Figures 6a and 6b present the quantified importance of dose-volume features within the learned embedding space, as determined by the AGM method. Figure 6a shows the overall contribution of each feature across all samples, while Figure 6b highlights feature importance separately for ORN-positive and ORN-negative cases.

Across both analyzes, features in the D30%–D65% range exhibit the highest importance, with D30% and D40% being the most influential overall. For ORN-positive cases, D30%, D35%, D40%, D50%, D55%, D60%, and D65% contribute most strongly, whereas for ORN-negative cases, D30% to D65% show the highest importance. These patterns suggest that mid-to-high volume doses play a critical role in shaping the embeddings and may be particularly informative for distinguishing between ORN outcomes.

## 7 Discussion

In this study, we introduced *SC–NTCP*, a novel framework that leverages supervised contrastive learning to develop Normal Tissue Complication Probability (NTCP) models for predicting osteoradionecrosis (ORN) from dose-volume histogram (DVH) data. Our approach addresses fundamental challenges in DVH-based modeling including high dimensionality, multicollinearity, and poor inherent class separability by learning discriminative latent representations optimized for both contrastive discrimination and classification.

The proposed *SC–NTCP* framework demonstrated superior performance compared to conventional machine learning baselines across multiple evaluation metrics. The *SC–NTCP* model achieved the highest AUC outperforming logistic regression, deep MLP, CNN classifier, and SVM. Notably, the dose-only variant of our model (*SC–NTCP* (*No clinical*)), surpassed several baseline models that incorporated both dose and clinical features. This finding underscores the representational power of the learned embeddings, which capture clinically meaningful patterns that raw DVH features alone cannot adequately express.

The integration of clinical variables (dental extraction status and smoking history) with learned dose embeddings in the *SC–NTCP* model yielded the best overall performance, demonstrating that combining data-driven representations with established clinical risk factors provides a more comprehensive approach to ORN risk stratification. The model achieved a balanced F1-score of 0.4140, higher than all other models, indicating an improved ability to identify true positive cases without excessive false positives; a critical consideration in clinical decision-making, where both over- and under-prediction carry consequences.

Another key contribution of this work is the interpretability analysis, which provides insight into which dose-volume features most influence the learned representations. Through gradient-based attribution analysis (AGM), we identified that mid-to-high volume doses (D30%–D65%) exhibit the strongest influence on the embedding space, with D30%, D35%, D45%, D55%, D60%, and D65% showing the highest overall importance. This finding aligns with clinical observations that moderate-to-high dose exposure to substantial mandibular volumes is associated with increased ORN risk [22, 24].

Interestingly, the class-specific analysis revealed differential feature importance patterns between ORN-positive and ORN-negative cases. For ORN-positive patients, D30%, D35%, D40%, D55%, and D60% were the most influential, whereas for ORN-negative patients, D30% to D65% showed the highest importance. These differences suggest that the encoder learned to focus on distinct dose-volume characteristics when processing cases with different outcomes, potentially capturing subtle dosimetric patterns that distinguish high-risk from low-risk DVH profiles.

Our results compare favorably with existing NTCP models for mandibular ORN, which have similarly identified mid-to-high dose-volume levels as significant predictors of ORN [22, 24]. The proposed *SC–NTCP* model achieved competitive performance metrics while offering the added advantage of leveraging the complete DVH structure rather than relying on a single summary statistic, which enables more robust modeling.

Furthermore, the t-SNE visualizations (Figures 5a and 5b) reveal that the learned embeddings provide improved class separation relative to the original DVH feature space. This observation offers direct evidence that the contrastive learning objective transforms the initially overlapping DVH data into a better discriminative latent representation, thereby addressing a key limitation identified in prior studies [13].

This foundational work supports ongoing efforts to advance radiation-induced toxicity modeling, with the overarching goal of improving the quality of life for head and neck cancer patients [3, 4, 10, 25, 27]. As survival outcomes continue to improve, greater emphasis is being placed on understanding and mitigating treatment-related toxicities. These efforts aim to refine treatment strategies, reduce adverse effects, and ultimately enhance both patient wellbeing and long-term survival outcomes [4, 5]. Possible directions for future work include exploring alternative contrastive learning strategies, evaluating complementary interpretability techniques to further strengthen and validate the findings, and incorporating additional clinical variables to improve model robustness and clinical interpretability.

## 8 Conclusion

This study demonstrates that supervised contrastive learning offers a robust and principled approach to NTCP modeling for mandibular ORN from DVH data. By learning a latent space that maximizes class separability, the *SC–NTCP* framework overcomes challenges of high dimensionality, multicollinearity, and poor feature separability inherent in traditional models, enabling more accurate and interpretable ORN risk prediction.

This study is limited by its reliance on a single institutional dataset with a limited number of ORN-positive cases, which restricts the ability to evaluate predictions across individual ORN grades (I–IV). Future research will focus on incorporating the learned embeddings into other risk prediction models and applying this framework to other radiation treatment-associated toxicities that may potentially include multiple organs at risk.

## Figures and Tables

**Table 1:** Patient Characteristics and Distribution Across Demographic, Treatment, and ORN Outcome Variables

Covariate	Category	Count (%)
**Total**		1259 (100)
**Demographics**		
**Age**	Average (SD)	60.72 (10.07)
**Gender**	Female	215 (17)
	Male	1044 (83)
**Dental status**	No extraction	707 (56)
	Edentulous	210 (17)
	Extraction	342 (27)
**Smoking status**	Current	180 (14)
	Former	607 (48)
	Never	472 (37)
**Treatment Specifics**		
**Radiotherapy mandible dose**	Average (SD)	37.74 (12.51)
**Chemotherapy**	None	233 (19)
	Concurrent (conc)	624 (50)
	Induction + conc	285 (23)
	Induction	97 (8)
	Unknown	20 (2)
**Surgery**	Definitive	1043 (83)
	Postoperative	216 (17)
**Outcome**		
**ORN**	Positive	173 (13.7)
	Negative	1086 (86.3)

**Table 2:** Performance Comparison Between Baseline Models and the Proposed *SC–NTCP* Framework

Model	AUC (95% CI)	ACC (95% CI)	F1-score (95% CI)
LR	0.753 (0.061)	0.692 (0.022)	0.382 (0.060)
MLP	0.743 (0.051)	0.657 (0.029)	0.376 (0.044)
CNN	0.744 (0.039)	0.640 (0.045)	0.373 (0.031)
SVM	0.740 (0.054)	0.661 (0.032)	0.393 (0.041)
***SC–NTCP***	**0.772** (0.050)	**0.701** (0.022)	**0.414** (0.041)

## CCS Concepts

• Computing methodologies → Representation learning; Supervised learning.
